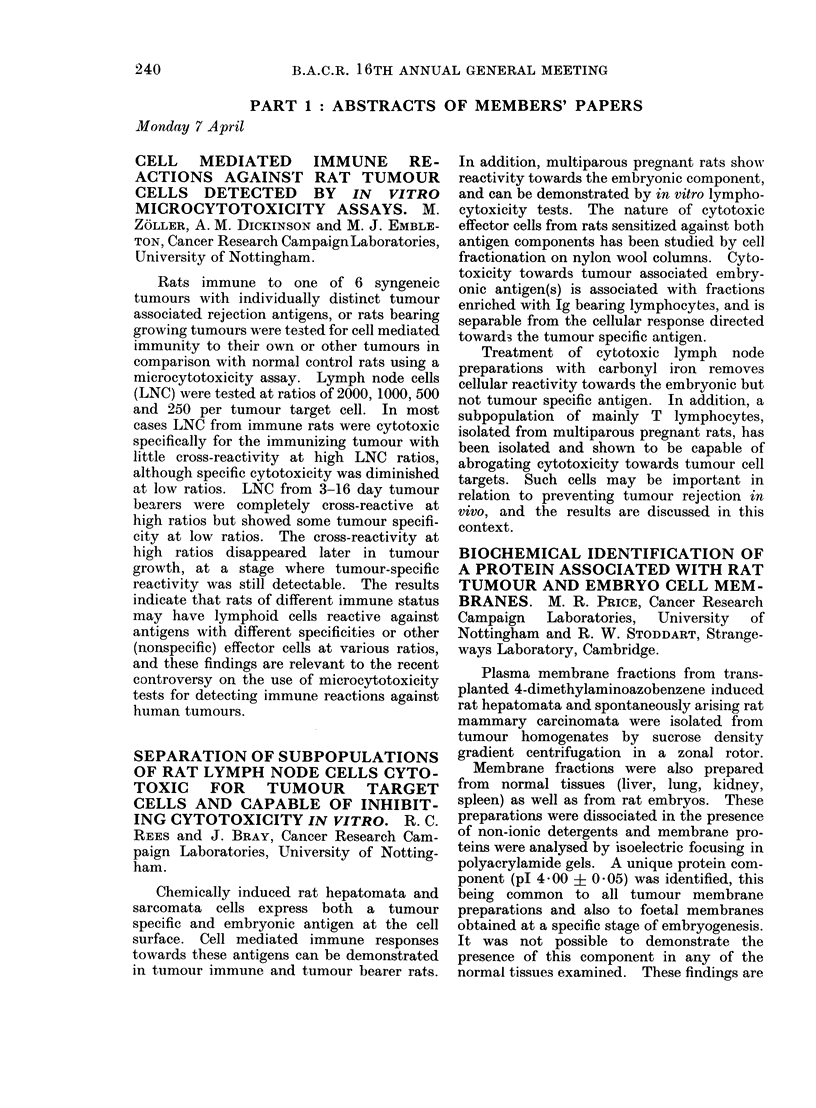# Proceedings: Cell mediated immune reactions against rat tumour cells detected by in vitro microcytotoxicity assays.

**DOI:** 10.1038/bjc.1975.154

**Published:** 1975-08

**Authors:** M. Zöller, A. M. Dickinson, M. J. Embleton


### PART 1 : ABSTRACTS OF MEMBERS' PAPERS

*Monday 7 April*

### CELL MEDIATED IMMUNE REACTIONS AGAINST RAT TUMOUR CELLS DETECTED BY *IN VITRO* MICROCYTOTOXICITY ASSAYS.

M. Zöller, A. M. Dickinson and M. J. Embleton, Cancer Research Campaign Laboratories, University of Nottingham.

Rats immune to one of 6 syngeneic tumours with individually distinct tumour associated rejection antigens, or rats bearing growing tumours were tested for cell mediated immunity to their own or other tumours in comparison with normal control rats using a microcytotoxicity assay. Lymph node cells (LNC) were tested at ratios of 2000, 1000, 500 and 250 per tumour target cell. In most cases LNC from immune rats were cytotoxic specifically for the immunizing tumour with little cross-reactivity at high LNC ratios, although specific cytotoxicity was diminished at low ratios. LNC from 3–16 day tumour bearers were completely cross-reactive at high ratios but showed some tumour specificity at low ratios. The cross-reactivity at high ratios disappeared later in tumour growth, at a stage where tumour-specific reactivity was still detectable. The results indicate that rats of different immune status may have lymphoid cells reactive against antigens with different specificities or other (nonspecific) effector cells at various ratios, and these findings are relevant to the recent controversy on the use of microcytotoxicity tests for detecting immune reactions against human tumours.